# Quality of care in patients with type 1 diabetes during the COVID-19 pandemic: a cohort study from Southern Brazil

**DOI:** 10.1186/s13098-022-00845-6

**Published:** 2022-05-21

**Authors:** Luciana Foppa, Janine Alessi, Betina Nemetz, Rosimeri de Matos, Gabriela Heiden Telo, Beatriz D. Schaan

**Affiliations:** 1 Hospital de Clínicas de Porto Alegre (HCPA), Rua Ramiro Barcelos 2350, Porto Alegre, RS 90035-003 Brazil; 2 Universidade Federal do Rio Grande do Sul, Rua Ramiro Barcelos 2400, 2º andar, Porto Alegre, RS 90035-003 Brazil; 3 Nurse School, Universidade Federal do Rio Grande do Sul, Rua Ramiro Barcelos, 2400, Porto Alegre, RS 90035-002 Brazil; 4 Hospital São Lucas da Pontifícia Universidade Católica do Rio Grande do Sul, Avenida Ipiranga, 6680, Jardim Botânico, Porto Alegre, RS 90619-900 Brazil; 5 Pontifícia Universidade Católica do Rio Grande do Sul, Avenida Ipiranga, Partenon, Porto Alegre, RS 668190619-900 Brazil

**Keywords:** Diabetes mellitus, Type 1, Quality indicators, Health care, COVID-19, Ambulatory care

## Abstract

**Background:**

Optimal glycemic control is the main goal for patients with diabetes. The results of type 1 diabetes patients’ neglected demands during the pandemic can determine a long-term negative clinical, social, and economic impact, and result in worse diabetes control and a higher incidence of chronic complications. Therefore, this study aims to evaluate the impact of the COVID-19 outbreak in the quality of care of patients with type 1 diabetes in Southern Brazil.

**Methods:**

Cohort study based on electronic medical records of patients with type 1 diabetes, with scheduled appointments between January 1st 2020, and November 6th 2020, at a university public hospital. The quality indicators used were: assessment of albuminuria and/or serum creatinine, lipid profile, thyroid-stimulating hormone, glycated hemoglobin, retinopathy, and neuropathy. McNemar test was used to analyze categorical variables and the Wilcoxon test for continuous variables.

**Results:**

Out of 289 patients, 49.5% were women aged 40 ± 12 years old. During the pandemic, 252 patients had at least one face-to-face appointment canceled. The quality of care indicators showed a significant worsening during the COVID-19 pandemic compared to the previous year (p < 0.001). In 2019, 23.2% of the participants had all the indicators evaluated, while in 2020, during the pandemic, only 3.5% had all of them evaluated.

**Conclusion:**

The COVID-19 pandemic hindered the offer of comprehensive and quality care to patients with type 1 diabetes.

## Introduction

The incidence of type 1 diabetes mellitus has progressively increased worldwide. In South and Central America, the number of adults with diabetes is predicted to rise to 49 million by 2045, an increase of 50% [1]. Brazil is the sixth country in absolute numbers of cases in adults in the world, and third for estimated number of new cases of type 1 diabetes [1]. Optimal glycemic control is the main goal for these patients, as it is associated with less acute and chronic diabetic complications [2, 3]. However, some challenges emerge, including the need for patient compliance with treatment, frequent blood glucose monitoring, attention to diet and physical activity and the risk of suffering from adverse treatment effects, especially hypoglycemia [4]. As a result, many subjects with type 1 diabetes fail to achieve optimal therapeutic targets [5, 6]. Goals setting can be useful to standardize the management of these patients and to provide multidisciplinary comprehensive care [7]. These goals may have a great impact in quality of care improvement, and also cover the clinical domains of diagnostics, monitoring, treatment and complications screening in individuals with type 1 diabetes [8, 9].

Coronavirus disease 2019 (COVID-19)—a disease caused by the SARS-CoV-2 started in 2019 in Wuhan, China—was declared by the World Health Organization (WHO) as a public health emergency of international concern on January 30th, 2020 [10]. In Brazil, although the first case of COVID-19 was confirmed on February 26, 2020, by July 2021 the mark of 19 million cases and 550 thousand deaths was surpassed, being considered one of the epicenters of the world pandemic [11, 12]. The social distancing measures affected the lifestyle of the population, brought changes in daily habits and health consequences, especially to people with type 1 diabetes [13, 14]. The practice of physical activities and the eating habits have been modified during the pandemic, affecting the control of the disease and may even worsen glycemic control [15, 16]. Moreover, the cancellation of medical appointments and the difficulty to obtain medicines and medical prescriptions may become a challenge to control this disease during the pandemic. These factors may negatively affect the quality of care for patients with type 1 diabetes, who may lose their routine monitoring and screening during the pandemic, possibly resulting in worse long-term outcomes.

Studies carried out to the date show that the COVID-19 pandemic and the social distancing measures have negatively impacted the results of the follow-up, control, screening, and vaccination indicators for diabetes patients in primary care centers [17, 18]. The pandemic has been increasing the prevalence of emotional stress, eating disorders, and sleep disorders of patients with type 1 and type 2 diabetes mellitus [14, 19]. However, no study performed to date has assessed how the current situation affects the quality of care for patients with type 1 diabetes, who need regular monitoring in specialized centers. During the pandemic, the results of these patients’ neglected demands can determine a long-term negative clinical, social, and economic impact, and result in worse diabetes control and a higher incidence of chronic complications. This study aims to evaluate the impact of the COVID-19 outbreak in the quality of care of patients with type 1 diabetes mellitus in Southern Brazil, according to the indicators proposed by guidelines.

## Methods

### Study design and setting

This is a cohort study of subjects with type 1 diabetes from Southern Brazil, with data collected retrospectively from medical records. Participants with a previous diagnosis of type 1 diabetes, with regular follow-up at the endocrinology outpatient clinic of a tertiary care public hospital in Southern Brazil were selected by requesting a query in electronic medical records with keywords, identifying all patients with type 1 diabetes mellitus treated in the institution. Electronic medical records were used to select participants who met the inclusion criteria for the study. Scheduled appointments between January 1st and November 6th, 2020, were used to assess the impact of the pandemic on quality of care indicators in this population, in comparison with the data collected from the same group of patients in 2019. In Brazil, the first case of COVID-19 was diagnosed on February 26th, 2020 [11], and, during the pandemic, three main waves occurred in the months of August 2020, December 2020 and March 2021 in the South region [20]. In March 22nd, 2020, the city of Porto Alegre, where most studied participants reside, presented its first requirement that guides social distancing and regulates establishments. Despite flexibility of social restrictions at some points, the social distancing for high risk groups for severe COVID-19, such as diabetic patients, remained very prevalent throughout 2020 [21].

### Participants

All patients aged over 18 years diagnosed with type 1 diabetes mellitus, who received outpatient care between January 2019 and November 2020 in our institution, were potentially eligible. For inclusion in the study, these patients should have at least one appointment scheduled between January and December 2020, regardless of whether this medical appointment was attended or not. Exclusion criteria were having a record of other types of diabetes—type 2 diabetes, MODY, LADA or uncertain type of diabetes—,pregnancy, death, outpatient discharge in 2019, or not having an outpatient appointment scheduled for the year of 2020.

### Data collection

To assess the impact of the pandemic on the quality of care indicators for type 1 diabetes, an evaluation of clinical and laboratory parameters of the same cohort of patients was conducted, from the research conducted in 2019, comparing the care provided during the pandemic COVID-19 in the year 2020. The information was collected for one year of follow-up before the last medical appointment in the period; these data were collected from the electronic medical records.

In order to identify possible flaws and to reduce bias, the researchers performed simulations and then collected data. The registering procedures were performed using an online form. The sociodemographic characteristics included age, sex, race/ethnicity and scholarity. The comorbidities were evaluated based on records of cardiovascular events, dyslipidemia, arterial hypertension, nephropathy, neuropathy, foot injuries, amputations and psychiatric conditions.

Furthermore, information regarding health appointments were obtained, including information on the number of outpatient visits (medical, nutrition and nursing), number of teleconsultations performed during the COVID-19 pandemic, body mass index (BMI), and blood pressure. The assessment of neuropathy was performed through the 10 g Semmes Weinstein monofilament evaluation, or the Ipswich Touch Test, or the vibration sensitivity evaluation records. The assessment of retinopathy was performed according to the last fundus examination or retinography recorded. The information registered was, preferably, extracted from the last appointment (medical, nutrition and nursing) of the patient at the institution.

Finally, laboratory results were collected, including the measurements of glycated hemoglobin (HbA1c, measured with high performance liquid exchange chromatography), creatinine, albuminuria, lipid profile (total cholesterol, LDL-cholesterol and triglycerides) and thyroid-stimulating hormone. These tests were performed at the laboratory division of the institution where the research was conducted. When there were more tests than recommended by the guidelines [4, 22], the last ones performed were considered.

### Quality of care indicators and outcome measures

The quality of care indicators chosen followed the guidelines of the American Diabetes Association and the Brazilian Diabetes Society [4, 22] and included:

1. HbA1c assessment: at least two annual measurements;
2. Retinopathy assessment: annually after 5 years of diagnosis;
3. Assessment of distal symmetric diabetic neuropathy: annually, using the 10 g Semmes–Weinstein monofilament or Ipswich Touch Test or assessment of vibration sensitivity;
4. Evaluation of albuminuria and / or serum creatinine: having at least one measurement in the last year;
5. Lipid profile (total cholesterol, HDL cholesterol, LDL cholesterol, and triglycerides): having a measurement in the last 3 three years;
6. Thyroid-stimulating hormone (TSH): a measure in the last 2 years;

Furthermore, it was checked if a glycemic target was registered in the patient’s online medical record. In daily clinical practice, patients who had a history of ischemic heart disease, records of frequent episodes of hypoglycemia, severe visual impairment, who underwent hemodialysis or peritoneal dialysis, and performed only two or less capillary blood glucose tests per day are considered for a flexible target (HbA1c ≤ 8.0%). For all other patients, strict glycemic control was considered adequate (target of HbA1c ≤ 7.0%).

The primary outcome was the presence of quality of care indicators (positive or negative) regarding the periods before and during the COVID-19 pandemic in the same cohort of patients. Secondary outcomes include the comparison between the two periods in relation to the presence of all minimum indicators of quality of care in diabetes and of each quality indicator individually, HbA1c levels, and lipid profile.

### Statistical methods

Sample size was calculated in the Power and Sample Size Health online version [23] to compare the percentage of individuals who had achieved the goal for quality indicators in 2019 but not in 2020, to the percentage of individuals who had not achieved the goal in 2019 but did in 2020. Considering 80% power and 5% significance level, the sample size necessary was at least 81 individuals [24].

Data analysis was performed by using the Statistical Package for Social Science (SPSS) version 22.0. Descriptive data are presented as frequency (%) or mean ± standard deviation (SD) if the assumption of normal distribution did not seem violated; otherwise, data were reported as median ± interquartile range (IQR). Normality was defined by the Shapiro–Wilk test. For the assessment of the primary outcome, a dichotomous outcome (yes/no) was considered for the presence of quality indicators previously defined. The difference between the two periods (prior to and during the COVID-19 pandemic) was assessed using the McNemar test for categorical variables and the Wilcoxon test for continuous variables. For association variables, Yates’s correction for continuity or Mann–Whitney U tests were used. An alpha value < 0.05 was used to determine statistical significance.

An exploratory post hoc analysis was performed comparing patients who were included in the study in relation to those excluded for not having a medical appointment scheduled during the pandemic period. The two groups were compared for age, gender, time of diagnosis of diabetes, HbA1c, and number of patients with glycemic control on the target.

### Ethical aspects

The study was approved by the institution’s Research Ethics Committee (No. 20380919800005327). The researchers signed a term of commitment for the use of the institution’s data, and the research considered the announced prerogatives in Resolution 466/2012 of the National Health Council. This document follows the STROBE Statement Checklist of items that should be included in reports of observational studies.

## Results

**Fig. 1:**
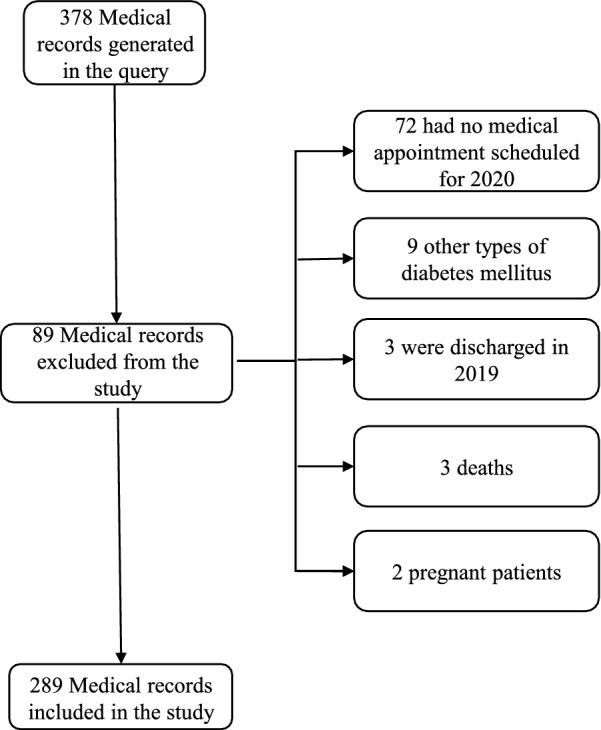
Flow diagram of exclusion criteria

After the initial identification, we obtained 378 medical records from patients with type 1 diabetes mellitus aged 18 years or older, who received care between January 2019 and November 2020. Out of these, we excluded 89 patients according to the exclusion criteria detailed in Fig. 1. In total, 289 patients were included in this study, mean age of 40 ± 12 years, and 143 (49.5%) were female. The other demographic and clinical characteristics are summarized in Table 1. During the pandemic, fasting blood glucose levels were lower (p < 0.001) compared to the period before the pandemic: 197 (133–260) mg/dL vs. 157 (99–236) mg/dL.

**Table 1:** Baseline demographic and clinical characteristics of the included participants (n = 289)

Patient characteristic	Female n = 143	Male n = 146	p-value^a^
Age (years)	38 (30–50)	39 (32–50)	0.340
Diabetes duration (years)	21 (14–30)	25 (15–31)	0.271
BMI (kg/m^2^)	24.8 (22.5–27.3)	24.8 (22.7–27.9)	0.680
Blood pressure (mmHg)			
Systolic	120 (110–130)	120 (115–138)	0.014
Diastolic	70 (70–80)	80 (70–80)	0.018
Chronic complications of diabetes n (%)			
Retinopathy	68 (47.6)	78 (53.4)	0.379
Nephropathy	24 (16.9)	27 (18.6)	0.821
Neuropathy	21 (14.8)	29 (20.1)	0.300
Limb amputation	2 (1.4)	6 (4.1)	0.296
Foot injuries	6 (4.2)	18 (12.3)	0.022
Macrovascular complications	12 (8.4)	13 (8.9)	1.000
Smoker n (%)	12 (8.4)	15 (10.3)	0.714
Dyslipidemia	19 (13.5)	21 (14.6)	0.921
Psychiatric disease	52 (37.1)	17 (12.3)	< 0.001
Arterial hypertension	37(25.9)	50 (34.2)	0.155

Data presented as number (%) for categorical variables or median and interquartile range for continuous variables

^a^The p-value is given for the Yates’s correction for continuity test for categorical variables and the Mann–Whitney U test for continuous variables. BMI: body mass index. Macrovascular complications included stroke, ischemic heart disease and peripheral arterial disease

The exploratory post hoc analysis comparing the baseline characteristics of the excluded patients for not having a medical appointment scheduled in the pandemic period, showed that there was no difference between the groups in terms of personal characteristics and glycemic control (data not shown).

During the pandemic, 252 patients had at least one face-to-face medical, nutrition or nursing appointment canceled. Considering the restrictions of conducting those outpatient visits and the Brazilian normative for telehealth in different areas, teleconsultations were introduced in the care of patients, including 98 appointments that were performed by physicians, 6 by nutrition teams and 14 by nursing teams. Furthermore, 10 patients received care by two or more professionals from the multiprofessional team. Only 28 patients did not have any type of assistance—face-to-face nor teleconsultation—in 2020.

The number of appointments with the multiprofessional team received by the participants during the COVID-19 pandemic was lower as compared with the period before the pandemic (p < 0.001). In 2019, considering face-to-face and teleconsultation appointments the median number of medical appointments performed was 4.0 (3.0–5.0), and during the pandemic (2020), the median was 2.0 (1.0–3.0). The participants had a median of 1.0 (0.0–2.0) face-to-face appointments with the nutrition team in 2019, in contrast with 0.0 (0.0–1.0) in 2020 (face-to-face and teleconsultation appointments). Furthermore, they had a median of 0.0 (0.0–1.0) consultation with the nursing team in 2019 (only consultation face-to-face), in contrast with 0.0 (0.0–0.0) in 2020 considering face-to-face and teleconsultation appointments.

The analysis of the quality of care indicators showed a significant worsening during the COVID-19 pandemic compared to the previous year. There was a reduction in the number of participants who presented all the indicators evaluated, according to the guidelines, in relation to the period prior to the pandemic. Moreover, the reduction in the total number of indicators was significant (p < 0.001): in 2019, 67 (23.2%) participants had all the indicators evaluated, whereas in 2020, during the pandemic, it was only 10 (3.5%). The number of patients who had each of the quality of care indicators evaluated, in the two periods evaluated, are described in Table 2. There was no difference in mean HbA1c, albuminuria and triglyceride levels in both periods. Considering achievement of glycemic targets, in the year of 2019 26% (n = 75) of the participants whose HbA1c target could be flexible were on the target, and only 1% (n = 3) of those whose HbA1c target should be strict were on the target.

**Table 2:** Number of patients who presented the quality of care indicators for type 1 diabetes mellitus according to the guidelines in the two periods evaluated (n = 289)

Quality of care indicator	2019	2020	P-value*
Assessment of distal simetric diabetic neuropathy	152 (52.8)	119 (41.3)	0.004
Assessment of albuminuria and / or serum creatinine^a^	247 (86.1)	206 (71.8)	< 0.001
Albuminuria (mg)	6.0 (3.0–18.0) 0.9 (0.7–1.0)	6.0 (4.0–14.0) 1.0 (0.8–1.2)	0.93 < 0.001
Mean creatinine (mg/dl)			
HbA1c^a^	263 (91.6)	135 (47.0)	< 0.001
Number of exams done	3 (2–3)	1 (1–2)	< 0.001
HbA1c value (%)	8.6 (7.8–9.8)	8.6 (7.6–9.6)	0.12
HbA1c value (mmol/mol)	70.0 (62.0–84.0)	70.0 (60.0–81.0)	
Lipid profile^a^	242 (84.3)	266 (92.7)	< 0.001
Total cholesterol (mg/dl)	173.0 (151.0–200.0)	178.0 (152.0–209.0)	< 0.001
HDL cholesterol (mg/dl)	56.0 (46.2–67.0)	53.0 (45.0–64.2)	< 0.001
LDL cholesterol(mg/dl)	101.0 (77.3–124.5)	104.8 (82.5–128.9)	< 0.001
Triglyceride (mg/dl)	78.5 (58.8–112.0)	81.0 (60.5–108.0)	0.39
Retinopathy assessment^b^	172 (63.9)	83 (30.9)	< 0.001
Thyroid-stimulating hormone assessment	262 (91.6)	256 (89.5)	0.080

Data presented as number (%) for categorical variables or median and interquartile range or mean and SD for continuous variables

*HbA1c* glycated hemoglobin, *HDL* high density lipoproteins, *LDL* low density lipoproteins

^a^Number of tests assessed and median values based on the number of patients who presented quality of care indicators each year

^b^Five years of diagnosis: n = 271

^*^The p-value is given for the McNemar test for categorical variables and the Wilcoxon test for continuous variables

## Discussion

Our study assessed the impact of the COVID-19 outbreak on quality of care parameters in a cohort of patients with type 1 diabetes in Southern Brazil, according to the indicators proposed by the guidelines. The evaluation of the same cohort of patients at two different times, before and during the outbreak, showed a negative impact of the pandemic on the care of type 1 diabetes. The analysis of quality of care indicators revealed a significant worsening during the pandemic compared to the previous year. The number of patients who had all the indicators evaluated was reduced compared to the period before the pandemic, however, there was no difference in participants’ glycemic control between the two evaluated periods.

The analysis of the quality of care indicators showed a significant worsening in the frequency of physician, nutrition and nursing appointment, and in the quality parameters when comparing the periods before and during the COVID-19 pandemic. Most evaluated patients did not receive face-to-face care with multidisciplinary teams during the pandemic. These results were expected in the context of the outbreak, since the population was instructed to stay at home and social restrictions were observed in several countries, including the reduction in scheduled appointments [25, 26]. Individuals with chronic diseases, such as diabetes, have been the most affected by these measures [27]. Moreover, patients who are afraid of going to the hospital made it difficult to maintain a regular assessment routine for diabetes care during the pandemic [28, 37]. Our study also showed a worsening in the participants’ lipid profile, which may be related to worsening eating habits and lower frequency of physical activity during the pandemic [16, 19].

Since the beginning of the pandemic, hyperglycemia has been associated with worse outcomes in COVID-19 infection, which can generate fear and concern to patients [28–31]. As a result, patients had the preference and were recommended to postpone their clinic visits. The use of multidisciplinary teleconsultations was one of the alternatives adopted to alleviate these assistance difficulties nationally and internationally [26, 32, 33]. Remote technology consultations are increasingly integrated into diabetes care and offer the opportunity to maintain continuity of care in a pandemic setting [19, 31, 34]. However, this approach has drawbacks, especially among patients and healthcare professionals that are not used to it, and also considering the rapid modification that usual care has turned to this new manner. It may exclude disadvantaged and vulnerable patients most in need of support, as disparities in access have been consistently shown, and may disproportionately benefit patients with more resources who already have support for their care, leading to intervention-generated inequalities [35]. As strategies to maintain the quality of care, in addition to teleconsultation, we can mention three which could be useful. Firstly, teleeducation and the online submission of educational folders, explaining the importance of maintaining self-care measures, even when elective care is suspended. Secondly, telementoring or creating groups in text message apps guided by a health care professional could be initiatives, so that the main difficulties experienced can be heard and proposals provided to help remotely. Thirdly, creating homecare groups (voluntary or not) in emergency situations, for the collection of exams at home and the evaluation of situations that need brief intervention.

The consequences of the social distancing measures, unavailability of health care appointments and worsening of the diabetes quality of care have the potential to generate negative impacts on patients with type 1 diabetes. Firstly, it is possible that the worsening of the quality of care parameters results in worse glycemic control, increasing the risk of serious decompensation and complications of the disease. Even before the pandemic, the quality of care for patients with type 1 diabetes in Brazil was already being evaluated, and it was identified the need to improve care for these patients [36].

Our study showed no difference in participants’ glycemic control comparing the periods before and during the COVID-19 pandemic. However, the data were analyzed only for the percentage of patients who collected exams in 2020, making it impossible to predict whether the missing data would represent patients with worse glycemic control, because of less assistance, for example, or better glycemic control, because they were more at home taking care of themselves, for example. The comparison of patients evaluated in 2020 with those who were excluded from the study because they did not have an appointment scheduled during the pandemic showed no differences. Ghosal et al. designed a mathematical model to demonstrate the possible impact that social isolation and lockdown measures may have on glycemic control in patients with diabetes and predicted a significant increase in HbA1c and future complications related to diabetes [37]. Furthermore, the lack of follow-up with multidisciplinary teams may result in loss of the patient’s bond with the health service, which may not return to the previous care routine after the end of the pandemic. Moreover, these factors directly affect the mental health of these patients, who are at a higher risk of presenting depressive symptoms, diabetes-related emotional distress, eating, and sleeping disorders [14, 38]. These points justify the importance of maintaining minimum parameters of care during periods of pandemic, and reflect the need to develop and to strengthen alternative care measures in these scenarios.

This study has some limitations, as based on medical records review, it is possible that there are biases related to the recording of information. Besides, the limitations inherent to the design of a cross-sectional study have to be considered, not being possible to attribute causal relationships to the associations found. Another aspect that should be considered is that the use of telehealth appointments was not a routine in our hospital at the beginning of the pandemic, it was gradually introduced from May 2020, when health professionals were trained and a specific telehealth system was developed. Thus, it is possible that, in a system in which telehealth appointments are already practiced, the impact of the pandemic on parameters of quality of care in diabetes would be less significant. Even so, it has to be considered that it was the reality experienced in developing countries in 2020, and health professionals should be alert to the consequences that will be experienced in the coming years.

The COVID-19 outbreak impacted significantly worsening the quality of care indicators for type 1 diabetes compared to the pre-pandemic period in the surveyed patients. This may reflect the difficulties faced during the pandemic to offer integral and quality care to these patients, and also the difficulties for patients in maintaining their self-care while not entirely assisted by health care professionals. Our data suggests that, in the first year of the pandemic, we were unprepared to provide support for patients with type 1 diabetes and drew attention to the need for strategies to maintain the quality of care in similar situations. Different alternatives may be useful to mitigate the effects of health care appointments interruption in the future, such as the use of information and communication technology, the organization of a self-care plan that does not depend directly on face-to-face appointments and the organization of a homecare service, capable of attending and arranging laboratory tests for specific cases. Even so, it is still necessary that specific protocols are developed, and resources are allocated to the organization of these measures, which need to be elaborated and programmed in advance in order to assist these patients timely.

## Data Availability

The dataset used and/or analyzed during the current study are available from the corresponding author on reasonable request.

## Abbreviations

BMI: Body Mass Index
COVID-19: Coronavirus disease 2019
HbA1c: Glycated hemoglobin
HDL cholesterol: High density lipoprotein
IQR: Interquartile range
LADA: Latent autoimmune diabetes in adult
LDL cholesterol: Low density lipoprotein
MODY: Maturity onset diabetes of the young
NGSP: National glycohemoglobin standardization program
SARS-CoV-2: Severe acute respiratory syndrome coronavirus 2
SD: Standard deviation
SPSS: Statistical package for social science
TSH: Thyroid-stimulating hormone
USA: United States of América
WHO: World health organization
